# 

**DOI:** 10.7189/jogh.10.010323

**Published:** 2020-06

**Authors:** Marni Sommer, Garazi Zulaika, Margaret L Schmitt, Samantha Khandakji, Penelope A Phillips-Howard

**Affiliations:** 1 Department of Sociomedical Sciences, Mailman School of Public Health, Columbia University, New York, New York, USA; 2 Department of Clinical Sciences, Liverpool School of Tropical Medicine, Liverpool, UK

The issue of menstrual health has gained significant traction in recent years as a fundamental aspect of public health, with significant relevance in low- and middle-income countries (LMIC) [1]. Research, practice, and policy addressing menstrual hygiene management (MHM) engages a growing number of actors, including researchers, practitioners, donors, policy makers, social entrepreneurs, national governments, advocates, and civil society. Globally, these actors work to address the social, environmental, and political factors that reinforce menstruation-related challenges experienced by girls and women in varying contexts.

To date, the largest body of evidence in the space collectively referred to here as menstrual health and hygiene (MHH) originates from descriptive qualitative and quantitative studies among girls in school, exploring the barriers they face with the onset of menstruation [2,3]. These challenges often reflect the insufficient education and guidance girls receive prior to their first menstrual period (menarche) from their families, communities or the education system. Other barriers are tied to the on-going stigma and taboos relating to menstruation that reinforce the need for secrecy and silence on menstrual management and enforce behavioral restrictions around (1) sleeping arrangements, (2) engaging in prayers or household chores, and (3) school participation. Additionally, inadequate access to clean and safe toilets with water, lacking disposal mechanisms for used menstrual materials, and inadequate menstrual products and related supplies, such as underwear, leave girls with limited agency to manage their menstrual periods [3].

In recent years, additional evidence has emerged from pilot intervention trials conducted with schoolgirls that have assessed the impact of the delivery of menstrual products and information on sexual and reproductive health outcomes, and educational performance [4,5]. There is an urgent need to rigorously assess the impact of the many MHH interventions currently being deployed in numerous LMIC. Another emerging area is humanitarian contexts, with researchers and practitioners focusing on the MHH needs of the over 30 million internally displaced and refugee girls and women around the world, and how to more effectively deliver holistic MHH solutions in such contexts [6]. Additional MHH learnings are needed in countries and contexts where the menstruation-related challenges facing girls have not yet been explored, and although some studies have included the MHH needs of out of school girls [7] and girls with disabilities [8], these are areas in need of additional exploration.

## THE “MHM IN TEN” AGENDA AND BEYOND

One framework to support assessments focused on school girls is the “MHM in Ten” agenda, which through collective inputs identified a ten-year vision and five key priorities for transforming schools for menstruating girls (2014-2024) [1]. The midway point in the MHM in Ten agenda, 2019, provides an important opportunity to assess progress made across the five key priorities focused on improving school environments for both students and teachers, as well as identifying the menstruation-related needs of girls outside of school and improving measurement of interventions targeting those outside of the formal educational systems. An in-depth effort to review progress across these priorities is under way, with initial findings suggesting that progress has been achieved across Priorities 1, 2, and 3, with slower achievements found towards Priorities 4 and 5, (see **Box 1**).

Box 1 **Five priorities of the menstrual hygiene management (MHM) in Ten Agenda.**

**Priority 1**: Build a strong cross-sectoral evidence base for MHM in schools for prioritization of policies, resource allocation, and programming at scale.

**Priority 2**: Develop and disseminate global guidelines for MHM in schools with minimum standards, indicators, and illustrative strategies for adaptation, adoption, and implementation at national and subnational levels.

**Priority 3**: Advance MHM in schools activities through a comprehensive evidence-based advocacy platform that generates policies, funding, and action across sectors and at all levels of government.

**Priority 4**: Allocate responsibility to designated government entities for the provision of MHM in school, including adequate budget and monitoring and evaluation (M&E); and the reporting to global channels and constituents.

**Priority 5**: Integrate MHM and the capacity and resources to deliver inclusive MHM into the education system.

Priorities 4 and 5 are dependent on a number of factors, including political commitment to addressing MHH as a critical aspect of on-going gender discrimination in educational systems, and sufficient intervention evidence and measurement available to enable governments and donors to invest resources with confidence. In recognition of the measurement gap in the field of MHH at large, support was provided by the Water Sanitation Supply Collaborative Council (WSSCC), a UN agency, in March of 2019 to Columbia University and its partners with the aim of bringing together key measurement and monitoring experts from the critical relevant sectors to align outcome and impact measurement priorities. These key sectors or priority areas include Water, Sanitation and Hygiene (WASH), Education, Gender, and Health (sexual and reproductive / psychosocial). The meeting produced a “green paper” [9], which outlined the identified alignment of MHH, in relation to girls in and out of school, with the priority areas for global monitoring. The analysis also articulates the ways in which improved alignment across these key priority areas is central to achieving numerous Sustainable Development Goals (SDGs) tied to Health, Education, Gender, Sanitation and beyond.

In tandem with collaborations between governmental, non-governmental, and/or advocacy organizations, the emergence of numerous menstrual health innovations led by social entrepreneurial organizations has elevated the growing menstrual movement over the last decade. Seeking to address the challenges that girls and women face in accessing menstrual products and supporting information, innovations have reimagined both hygiene products and female-centered distribution models. Given the growing number and diversity of menstrual innovations being developed and/or delivered in LMIC, there is a need to better measure the impact of these innovations and their delivery, both to support their efforts and to inform government and donors seeking to invest their limited resources wisely.

**Figure:**
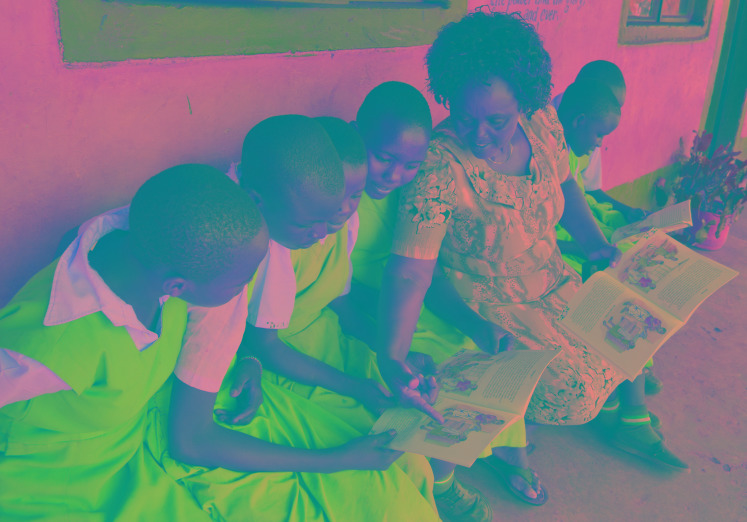
Photo: A school teacher in Kenya helps to prepare adolescent girls about menstruation and the onset of puberty (Plan International, 2019, used with permission).

## A THEORY OF CHANGE AND FRAMEWORK FOR MEASURING MHH INNOVATION

In an effort to improve implementation planning and evaluation of menstrual health innovations, a *Theory of Change (TOC) for menstrual health innovations and interventions* and accompanying Monitoring and Evaluation (M&E) framework was developed in 2017-2018 through support from Grand Challenges Canada (GCC). Although the original intention was to provide a TOC and M&E framework for GCC grantees focused on menstrual product innovation and information delivery at scale, the extensive nature of the undertaking resulted in the development of valuable resources for the larger global community implementing MHH interventions for girls and women [10].

The TOC schematic spans different stages of menstrual health innovation efforts, starting from pre-production and onward through the distribution of a variety of menstrual products (eg, reusable pads, disposable pads, menstrual cups), and dissemination approaches (eg, direct distribution, retail, through schools and/or NGOs). Consideration of distribution of information, communication and education (ICE) materials and/or awareness campaigns is also included, highlighting messaging around sexual and reproductive health, MHM, taboos and social norms. An accompanying narrative supports the TOC diagram, including more detailed information.

The M&E framework developed in parallel provides specific indicators for describing and quantifying changes that result from the development, introduction, and use of the MHM interventions following the TOC schematic. The aim of the framework is to enable innovator success in meeting targets and goals over time and to provide improved evidence to investors and policy makers, by using standardized indicators and measures. Collective learning from improved assessments will strengthen the evidence base on recommended indicators for the larger MHH community.

## THE WAY FORWARD

Much remains to be done in the realm of menstrual health and hygiene management to make progress on meeting the MHM in Ten vision and priorities in relation to transforming schools for menstruating girls by 2024, in addition to addressing the MHH issues faced by girls out of school, living in displacement contexts, and experiencing disabilities. Advocacy efforts for MHH continue to grow, the evidence base is expanding, and governments and policymakers are finally starting to take notice. Central to building on current support and advocacy is the improvement of measurement of interventions being delivered at the local, national, and international levels. The guidance offered through the MHM in Ten agenda, and the TOC and M&E framework for scaling menstrual health innovations, provide insights for a collective way forward.
